# Benefits of Chimeric Antigen Receptor T-Cell Therapy for B-Cell Lymphoma

**DOI:** 10.3389/fgene.2021.815679

**Published:** 2022-01-20

**Authors:** Wenyujing Zhou, Weihong Chen, Xiaochun Wan, Changru Luo, Xin Du, Xiaoqing Li, Qian Chen, Ruiwen Gao, Xiaohan Zhang, Mei Xie, Mingjun Wang

**Affiliations:** ^1^ Department of Hematology, The First Affiliated Hospital of Shenzhen University/Shenzhen Second People’s Hospital, Shenzhen, China; ^2^ Center for Protein and Cell-based Drugs, Institute of Biomedicine and Biotechnology, Shenzhen Institute of Advanced Technology, Chinese Academy of Sciences, Shenzhen, China; ^3^ Shenzhen BinDeBioTech Co., Shenzhen, China; ^4^ Research Management and Supporting Department, Shenzhen Institute of Advanced Technology, Chinese Academy of Sciences, Shenzhen, China; ^5^ Shenzhen Institute for Innovation and Translational Medicine, Shenzhen, China

**Keywords:** anti-CD19 CAR T-cell, CD19, relapsed/refractory B-cell lymphoma, benefit, CRS

## Abstract

**Objective:** The aim was to study the benefits and risks of anti-CD19 chimeric antigen receptor (CAR) T-cells in adults with B-cell lymphoma.

**Methods:** From October 2015 to October 2021, we treated five patients with B-cell lymphoma, comprising two with mantle cell lymphoma, one case of Burkitt lymphoma, one case of diffuse large B-cell lymphoma, and one case of chronic lymphocytic leukemia/small lymphocytic lymphoma. The patients were given the FC regimen 5 days before the infusion of anti-CD19 CAR T-cells. The median total number of CAR T-cells infusions was 350*10^6 (88*10^6–585*10^6).

**Results:** 1) Patients who received CAR T-cell induction therapy achieved complete remission (CR) in Case 1 and Case 3 and partial remission (PR) in Case 2. Case 3’s ATM and D13S25 gene deletions were negative 42 days after CAR T-cell therapy, and molecular biology CR (mCR) and minimal residual disease (MRD) were negative for 5 years and 6 months. The patient in Case 3 was cured. 2) Case 4 patient’s TP53 gene mutation became negative 1 month after CAR T-cell therapy. MRD was negative after CAR T-cell therapy at 41 and 42 months in Cases 4 and 5, respectively. 3) Case 1∼Case 3 patients developed cytokine release syndrome (CRS) without encephalopathy syndrome, accompanied with serious adverse events. CRS can be effectively managed with tocilizumab, etanercept, glucocorticoids, and plasmapheresis.

**Conclusion:** Anti-CD19 CAR T-cell therapy is effective in treating relapsed/refractory B-cell lymphoma, and the side effects of CAR T-cell therapy can be properly managed. CAR T-cell therapy has high efficacy and presented no side effects in the treatment of MRD in B-cell lymphoma (NCT03685786, NCT02456350).

## 1 Introduction

B-cell lymphoma is the most common type of malignant lymphoma. Relapsed/refractory (R/R) lymphoma is the main cause of treatment failure. In 2015, there were about 71,000 cases of non-Hodgkin lymphomas (NHLs) with approximately 19,700 deaths related to the disease. NHL is the seventh leading cause of new cancer cases (Cheson and Leonard, 2008). Till date, 30–50% of NHL patients are refractory to the standard treatment or relapse after remission. The prognosis of these patients is extremely poor, with a dismal objective response rate of 26% and a median overall survival of 6.3 months after salvage treatment (Coiffier and Sarkozy, 2016; Crump et al., 2017). Chimeric antigen receptor (CAR) T-cell therapy represents a novel and paradigm shift in the cancer treatment approach. The immunotherapy approach using genetically modified cytotoxic immune T-cells to target tumor-specific antigens has resulted in durable remissions in R/R B-cell lymphoma. Currently, there are two FDA-approved products for the treatment of R/R B-cell NHL namely tisagenlecleucel and axicabtagene ciloleucel (Davila et al., 2014a; Maude et al., 2015). Tisagenlecleucel is also approved for the treatment of relapsed and/or refractory pediatric B-ALL up to the age of 25 years. On February 5, 2021, the United States Food and Drug Administration (FDA) approved lisocabtagene maraleucel for the treatment of relapsed or refractory large B-cell lymphoma (FDA, 2021). Structurally, CAR T-cells are autologous T-cells that express cancer-targeted CAR through genetic engineering. The CAR molecule is composed of two parts: an antigen-recognizing extracellular domain, commonly a single-chain antibody fragment (scFv), and an intracellular signaling domain (Essand and Loskog, 2013; Dotti et al., 2014). The latter merges signaling domains from the T-cell receptor (TCR) complex and co-stimulatory protein, such as CD134/OX40 (Pulè et al., 2005) and CD137/4-1BB (Imai et al., 2004; Stephan et al., 2007; Carpenito et al., 2009).

## 2 Methods

### 2.1 Case and Data

Patients with R/R B-cell lymphoma admitted to the First Affiliated Hospital of Shenzhen University/Shenzhen Second People’s Hospital (Shenzhen, China) between October 2015 and October 2021 were selected based on WHO lymphoma classification criteria. The lymphoma was diagnosed in 2015 and was screened according to clinical trials inclusion and exclusion criteria. The patients with CD19-positive lymphoma cells were examined for pathological biopsy and immunohistochemistry of lymphoid tissue before chimeric antigen receptor T-cell (CAR T-cell) therapy. The patients in the group were fully aware of the clinical research and signed informed consent.

### 2.2 Clinical Characteristics

We collected the general information of patients included in this study, including gender, age, and disease and evaluated tumor burden of patients before CAR T-cell therapy. The characteristics of the five patients (Case 1∼Case 5) are shown in Table 1. The five patients were diagnosed with B-cell lymphoma, with ages ranging from 32 to 68 years, with a median of 60 years. Before receiving CAR T-cell therapy, all patients received 2–9 chemotherapy regimens. The median follow-up time was 36 months (Table 2). There are two cases of mantle cell lymphoma (MCL), one Burkitt lymphoma (BL), one DLBCL, and one CLL/SLL, with four males and one female. It is worth noting that three patients have a history of using ibrutinib. Case 3 relapsed after receiving ibrutinib for 10 months prior to CAR T-cell therapy and then discontinued the use. Case 4 continued to receive ibrutinib from 1 year prior to CAR T-cell therapy till date, and Case 5 only received ibrutinib for 4 months after CAR T-cell therapy. Additionally, four of the five patients had genetic abnormalities prior to CAR T-cell therapy (Table 1): In Case 3, the bone marrow biopsy showed ATM and D13S25 deletions; in Case 4, the bone marrow biopsy showed NOTCH1, TP53, and ATM mutations; in Case 5, tumor tissue showed only a C-MYC-positive, while gene mutation in bone marrow remained negative. In Case 1 and Case 3, treatment was carried out with anti-CD19 CAR T-cell-induced significant therapy. Although imaging examination revealed that patients of Cases 4–5 had achieved CR prior to CAR T-cell therapy, the molecular genetics did not achieve remission. Therefore, the therapeutic goal of the two patients was to eliminate MRD and maintain therapy.

**TABLE 1:** Patient characteristics before CAR T-cell therapy and dose of CAR T-cell reinfusion.

	Case 1	Case 2	Case 3	Case 4	Case 5
Sex	M	M	M	M	F
Age (Age of CAR T-cell therapy)	60/63	49	68	60	32
Diagnose	MCL	DLBCL (EB virus+)	CLL/SLL	MCL	BL
Tumor burden	The mass was 16 * 6.2 cm in the abdominal cavity, and multiple lymph nodes were enlarged, with the maximum diameter of 5.3 cm	Multiple lymphadenopathy with the largest diameter of 3.5 cm	Multiple lymphadenopathy with the largest diameter of 3.5 cm	Genetic mutations persist	Genetic mutations persist
Chromosome karyotype	—	—	Normal	Normal	Normal
Gene mutation	—	TET2 mutation	ATM and D13S25 deletion	NOTCH1, TP53, ATM mutation	C-MYC rearrangement
Therapeutic purposes	Induction therapy	Induction therapy	Induction therapy	Elimination of MRD and maintenance therapy	Elimination of MRD and maintenance therapy
Chemotherapy before CAR T-cell therapy	R-CHOP*3, CHOP*1	R-Hyper CVAD*1, BV-DICE*1, CHOP, EPOCH*1, EPOCH + L*1, EPOCH + Ara-c*1, GDPE*1, DHAP*1, MA*2	FCR*5, FR*1, BR*4	R-CHOP*3, CHOP*1	R-CHOP*1, R-IVCA*1, R + MTX*2, R-DHAOx*1, R-CODOX-M*3
Ibrutinib	No	No	Yes	Yes	Yes
Auto-HSCT	No	No	No	No	Yes
Allo-HSCT	No	No	No	No	Yes
ECOG performance-status score	2/2	2	2	1	1
β2-MG (ug/ml)	5.25/6.25	3.33	3.84	2.94	3.33
Number of CAR T-cell therapy courses	2	1	1	1	1
Infusion volume of viable CAR T-cells (10^6)	300/370	585	330	88	357

R-CHOP: rituximab, cyclophosphamide, Adriamycin, vincristine, and dexamethasone.

EPOCH: rituximab, etoposide, vincristine, pirarubicin, cyclophosphamide, and dexamethasone.

R-Hyper CVAD: rituximab, cyclophosphamide, pirarubicin, vindesine, and dexamethasone.

BV-DICE: rituximab, ifosfamide, bortezomib, carboplatin, and dexamethasone.

FCR: fludarabine, rituximab, and cyclophosphamide.

BR: rituximab and bendamustine.

R-DHAOx: dexamethasone, rituximab, cytarabine, and oxaliplatin.

R-CODOX-M: rituximab, ifosfamide, vindesine, pirarubicin, and MTX.

R-IVCA: rituximab, ifosfamide, etoposide, and cytarabine.

GDPE: gemcitabine, cisplatin, dexamethasone, and etoposide.

DHAP: cisplatin, Ara-c, and dexamethasone.

MA: MTX + Ara-c; MTX: methotrexate.

Ara-c: cytarabine; L: L-asparaginase.

MCL: mantle cell lymphomas; BL: Burkitt lymphoma; DLBCL: diffuse large B-cell lymphoma; CLL/SLL: chronic lymphocytic leukemia/small lymphocytic lymphoma; MRD: elimination of minimal residual disease.

**TABLE 2:** Value of measurable lymph nodes, extranodal lesions, and spleen (CT) (mm).

			Submandibular	Parotid gland	Supraclavicular	Cervical	Groin	Axillary	Mesentery	Abdominal cavity	Spleen
1	First course of treatment	Before CAR T-cell	20 × 9	15 × 6	30 × 12	32 × 13	53 × 22	27 × 11	—	160 × 62	—
		After CAR T-cell	9 × 4	8 × 4	12 × 7	14 × 5	27 × 10	14 × 4	—	48 × 15	—
	Second course of treatment	Before CAR T-cell	14 × 10	13 × 7	83 × 25	11 × 7	36 × 15	21 × 7	—	42 × 15	—
		After CAR T-cell	5 × 3	7 × 4	40 × 12	7 × 3	13 × 5	10 × 3	—	21 × 6	—
2		Before CAR T-cell	17 × 9	12 × 8	15 × 7	23 × 10	16 × 11	35 × 16	—	—	—	
		After CAR T-cell	14 × 7	15 × 9	9 × 8	26 × 11	40 × 12	20 × 14	—	—	—	
3		Before CAR T-cell	18 × 6	17 × 8	18 × 5	19 × 10	35 × 27	35 × 15	44 × 15	—	—	
		After CAR T-cell	<8	<8	<8	<8	<6	<6	<8	—	—	

-: normal; After CAR T-cell: 30 days after CAR T-cell infusion.

### 2.3 CAR T-Cell Therapy

#### 2.3.1 Pretreatment Scheme

FC scheme (fludarabine 25–30 mg/m^2^ × 3 days; cyclophosphamide 250–300 mg/m^2^ × 3 days).

#### 2.3.2 Treatment Process

The peripheral blood lymphocytes were collected and sent to the laboratory for genetic transformation into anti-CD19 CAR T-cells 1 month before transfusion. The patients received the FC regimen on the fifth day (day-5–3) before CAR T-cell infusion. CAR T-cells were reinfused into the patient on day 0–2 with 10, 30, and 60% of the anti-CD19 CAR T-cells consecutively. The non-steroidal anti-allergic drugs were used to avoid an allergic reaction. The antipyretic analgesic acetaminophen was administered to prevent fever before CAR T-cell infusion.

#### 2.3.3 Preparation and Reinfusion of CAR T-Cells

The median of the total number of reinfused CAR T-cells was 350*10^6 (88*10^6–585*10^6). The CAR T-cell expansion *in vitro* in Case 4 was relatively poor, and thus the dose of reinfusion is lower than that in others. The CAR T-cell reinfusion was only administered for two consecutive days, and all patients except Case 4 received three times of reinfusion in the first course of reinfusion. In addition, only one patient received the second course of CAR T-cell reinfusion due to relapse (Table 1).

#### 2.3.4 Testing Indicators

The serum cytokine levels and peripheral blood CAR levels were measured on days 0, 4, 7, 14, and 28.

The complete blood cell count (CBC), CD19, and IgG and other related indexes of peripheral blood were monitored every 3 days. The levels of CD19, CAR, and cytokines were detected every 3 months after the reinfusion of CAR T-cells. CT scans were performed on the 30th day after the reinfusion of CAR T-cells, every 3–6 months for the next 5 years, and every year after 5–10 years.

#### 2.3.5 CRS Treatment

The patients’ symptoms and syndromes, as well as their cytokines, CRP, and ferritin levels, were all monitored. The CRS was controlled by using tocilizumab and etanercept, alone or in combination. If the aforementioned treatment is ineffective, we try to control CRS by using glucocorticoids and/or plasmapheresis to reduce high levels of inflammatory factors and cytokines.

### 2.4 Reference Standard

CAR T-cell therapy was evaluated according to Lugano standard (2014). The diagnostic criteria for CRS are classified into four grades based on Lee scale, Penn scale, and CAR-TOX (Lee et al., 2014; Neelapu et al., 2018; Porter et al., 2018). We use the Penn scale to further divide the CRS into five grades, while other adverse reactions were assessed using The National Cancer Institute Common Terminology Criteria for Adverse Events (CTCAE 5.0).

## 3 Results

### 3.1 Tumor Burden (Short-Term Effects (<1 Month))

The short-term effect of CAR T-cell therapy was observed within 1 month in Cases 1–3, whose purpose was to induce treatment. The clinical effect of CAR T-cell therapy in Case 1 and Case 3 was relatively obvious, especially in Case 3 which attained CR in a short time with reduced tumor burden (long diameter of the lymph node was less than 1.5cm, no residual extranodal lesions, and the size of the spleen returns to normal). PR was obtained in the first and second courses of CAR T-cell therapy (the sum of PPD (longest diameter × short diameter perpendicular to the longest diameter) of six target lesions, that is, SPD (sum of product of vertical diameter of multiple lesions) ≥ 50%) in Case 1 (Table 2). In Case 2, there was no significant change in the lesions before and after treatment (Table 2). It should be noted, however, that the physical examination of Case 2 on day 14 showed that all of the enlarged superficial lymph nodes could not be touched. However, due to the rapid progression of the patient’s condition, he was unable to go to the ward for CT examination. In Case 4, the ATM, NOTCH1, and TP53 genes tested negative based on the next-generation sequencing (NGS) data 1 month after CAR T-cell therapy in bone marrow (Table 3).

**TABLE 3:** Result of CAR T patients.

	Case 1	Case 2	Case 3	Case 4	Case 5
Best response	CR	PR*	mCR	mCR	-
Gene mutation after CAR T-cell therapy	—	Persistent existence	Negative for ATM and D13S25 deletion	Negative for TP53, ATM, and NOTCH1 mutation	Persistent existence
CRS	2	3	2	No	No
CRES	No	No	No	No	No
PFS (month)	29/-^▲^	1.2	64	38	39
Current outcome	Survival	Death	Survival	Survival	Survival
Follow-up time	65	1.6	65	39	40

▲: Case 1: PFS was obtained in the first course of treatment for 29 months/unclear in the second course of treatment.

*:Case 2 had no objective imaging evidence of PR, but physical examination showed that all superficial lymph nodes retracted and could not be touched.

### 3.2 CAR T-Cell (Short-Term Effects (<1 Month))

**FIGURE 1:**
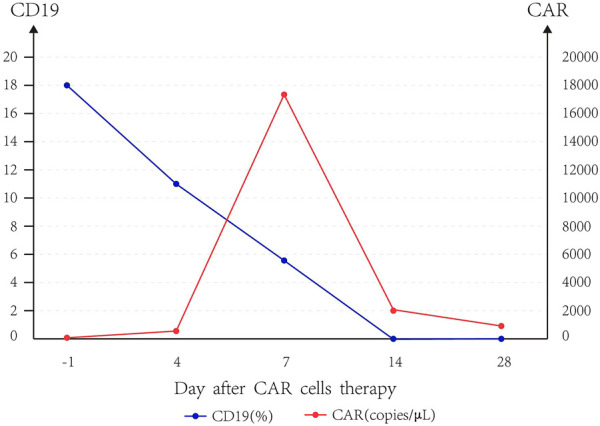
Trend of CD19 and CAR.

Taking the median of CAR and CD19 data of five patients, we can find that CAR copies peaked at day 7, and the expression of CD19 gradually decreased to 0 until day 14, and the slope of CD19 decline was the largest when CAR copies were the highest (Figure 1). The median amplification multiple of CAR was 16.94 (9.12, 249.3), and the median amplification duration was 7 days (2, 20).

### 3.3 Tumor Burden (Long-Term Effects Time(>1 Month))

The long-term therapeutic effect of patients treated with CAR T-cell therapy to induce remission is expected from 1 month after CAR T-cell therapy. The best response of patients was from Case 1 and Case 3 whose treatment induced complete remission. The Case 1 patient had got partial remission (PR) in a short time of the first course of CAR T-cell therapy and progressed after 29 months of PR for the first course of treatment. In the second course of CAR T-cell therapy, PR was achieved again in the short term, and CR was obtained in the first year of telephone follow-up. Unfortunately, due to the loss of follow-up, the specific time and duration of CR were unknown. More importantly, Case 3 not only achieved CR in the short-term efficacy assessment, but it also obtained negative ATM and D13S25 gene deletion results (molecular biology complete remission, mCR) at day 42 after CAR T-cell therapy. The Case 3 mCR has lasted 5 years and 6 months and is now considered clinically cured and alive. On the other hand, Case 2 progressed on the 35th day and died on the 47th day after CAR T-cell reinfusion.

Case 4 and Case 5 had MRD eliminated and maintained CAR T-cell therapy. ATM, NOTCH1, and TP53 mutation of Case 4 turned negative in the NGS result at 1 month after CAR T-cell therapy in bone marrow, and TP53 was still negative in FISH at day 180. At the end of follow-up for Case 4, NGS data still showed that ATM, NOTCH1 and TP53 were negative. Interestingly, Cases 4 and 5 were MRD negative at 41 and 42 months after CAR T-cell therapy, respectively (Table 3).

## 4 Toxicity and Treatment

### 4.1 CRS

CRS was found in the patients of Cases 1–3, including patients in Case 1 and 3 with CRS grade 2 and Case 2 patients with CRS grade 3. The patient of Case 1 was treated twice with CAR T-cells, with CRS grade 2 appearing in both treatment courses. The patients of Case 4 and Case 5, who had MRD eliminated and maintained therapy, did not have CRS (Table 1).

#### 4.1.1 Fever

**FIGURE 2:**
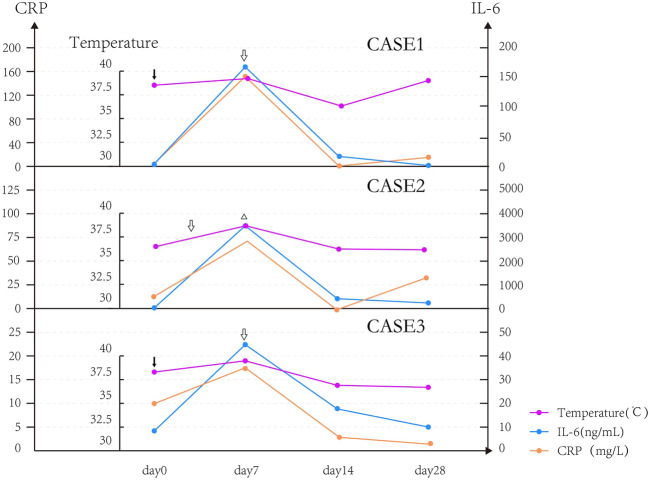
Changes in temperature and IL-6 after transfusion of CAR T-cells in patients with CRS.

All CRS patients had chills and fever. The body temperature of CRS patients began to drop on the seventh day after it exceeded 39°C, but they were unable to return to normal temperature and had repeated low fever, and the body temperature returned to normal after 1–2 weeks. We found that two patients with good curative effect (Case 1 and Case 3) had fever on the day of reinfusion, while the patient with poor curative effect (Case 2) had delayed fever and fever appeared on the seventh day. The patient’s body temperature gradually returned to normal after being treated with the recombinant humanized monoclonal antibody against human interleukin-6 (IL-6) receptor, tocilizumab, and glucocorticoids. In this study, we observed that the level of cytokines (IL-6) in Case 2 was significantly higher than those in Case 1 and Case 3. The body temperature and shock symptoms could not be effectively controlled after the use of tocilizumab. We used plasma exchange and glucocorticoids to reduce the high level of cytokines, as well as fluid replacement and dopamine, and achieved satisfactory results (body temperature returned to normal; shock was controlled). In addition, we also found that the trends in body temperature, IL-6, and CRP of three patients with CRS were similar and roughly parallel (Figure 2).

#### 4.1.2 Respiratory and Circulatory System

In Case 2 and Case 3 of hypotension to shock, one of them showed a decrease in blood oxygen saturation, and the aforementioned symptoms were relieved by using plasmapheresis after the poor effect of conventional support. Case 1 showed chest distress, shortness of breath, palpitation, and increased heart rate, and the symptoms were relieved after symptomatic and supportive care.

### 4.2 Other Adverse Events

#### 4.2.1 Hematological Changes

**FIGURE 3:**
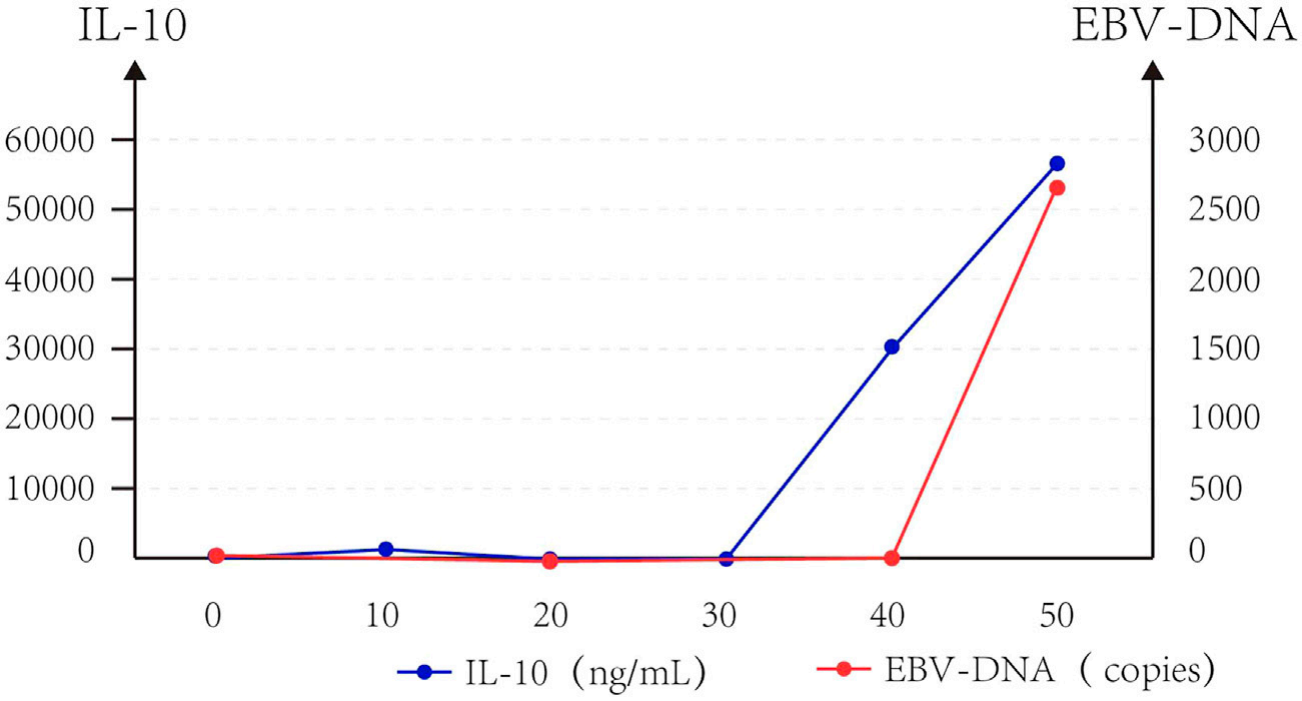
Trend of other indirect monitoring indicators.

Upon CAR T-cell therapy, two patients had leukopenia and neutropenia with fever, one patient had neutropenia with fever, and two patients had leukopenia without fever. Also, three patients had thrombocytopenia, and two patients had lymphocytopenia. Furthermore, platelets, leukocytes, and lymphocytes (median) decreased on day 0 and PLT gradually increased to normal on day 7, while leukocytes and lymphocytes gradually increased on day 14 (Figure 3).

#### 4.2.2 Non-Hematological Adverse Events

**FIGURE 4:**
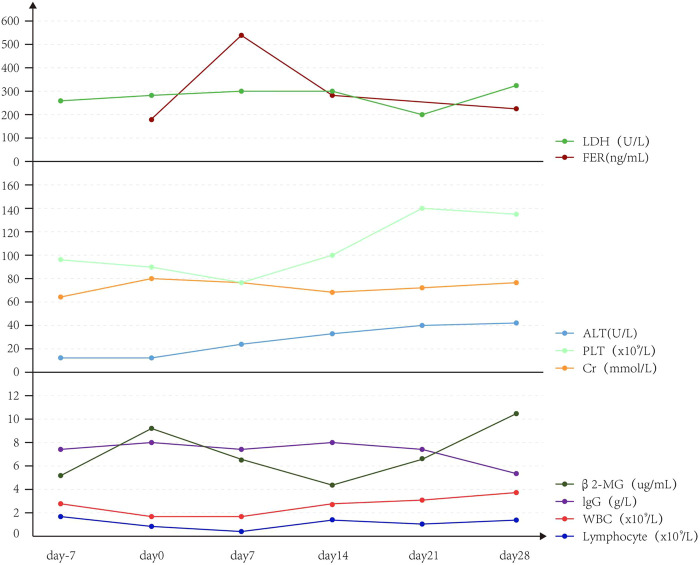
Trend of IL-10 and EBV-DNA copies of Case 2.

The patients had diarrhea, abdominal pain, abdominal distension, perianal burning, pain, oral ulcer, and other discomfort symptoms. The level of alanine aminotransferase (ALT) showed a slight upward trend, but imaging showed no obvious organic lesions in the liver and kidney (Figure 4). The adverse events associated with CRS grade 2 were significantly higher than those associated with CRS grade 1 or no CRS group (Table 4).

**TABLE 4:** Number of the patients of the adverse event.

	All	Grades 1 and 2	Grades ≥3	No CRS
Fatigue	3	1	2	0
Elevated BNP	2	1	1	0
Dyspnea	2	0	2	0
Diarrhea	1	0	1	0
Perianal pain	1	0	1	0
Mouth ulcer	1	0	1	0
Neutropenia	3	1	1	1
Leukopenia	5	1	2	2
Thrombocytopenia	3	0	1	2
Lymphopenia	2	0	1	1

At the same time, we observed that IL-10 and EB virus DNA copy increased approximately parallel after CAR T-cell infusion in Case 2 (Figure 3).

## 5 Discussion

Following a decade of preclinical optimization, anti-CD19 CAR T-cell therapy has produced impressive clinical results in patients with B-cell malignancy. CAR T-cell therapy is emerging as a promising therapeutic option on B-cell malignancies, with the potential for durable disease control following a single treatment. CAR T-cell therapy distinguishes itself from other therapies that require repeated and/or continuous administration. The previous NHL CR rate reported was 57.1% (Davila et al., 2014b; Kochenderfer et al., 2015). In this report, we demonstrated the potential of anti-CD19 CAR T-cell therapy for R/R B-cell lymphoma. Additionally, we demonstrated that the study’s objective response rate was 80% (4/5), while a CR rate of 66.6% (2/3) was observed. Interestingly, the gene deletion ATM and D13S25 which was initially positive for Case 3 patient resulted negative at day 42 post CAR T-cell therapy; also, after 1 month of CAR T-cell therapy, the TP53, ATM, and NOTCH1 gene mutations in Case 4 were found to be negative. Additionally, there are promising prospects in MRD elimination and maintenance therapy. Case 4 was MRD negative 41 months after receiving CAR T-cell therapy, while in Case 5 the progression-free survival (PFS) was 42 months. It should be noted that in this study patients with TP53 gene mutation turned negative only 1 month after receiving CAR T-cell therapy. The TP53 gene hot spot mutation is highly immunogenic and can trigger T cell responses to new antigens in tumors. Preliminary studies show that peripheral blood lymphocytes can recognize tumors with TP53 gene mutation after *in vitro* stimulation and *in vivo* immunization. All the above suggest that cancer patients with TP53 gene mutations may be more suitable for immunotherapy including CAR T-cell therapy used in this study. Although we use anti-CD19 CAR T-cells for treatment, the high-immunogenic response of TP53 gene mutation enhances the anti-tumor T-cell response of cancer patients. It improves the efficacy of anti-CD19 CAR T-cell-targeted therapy suggesting that the TP53 mutation could be a potential target of CAR T-cell therapy. We speculate that anti-CD19 CAR T-cells could have a therapeutic effect on TP53 gene mutations (Malekzadeh et al., 2019; Chasov et al., 2020; Titov et al., 2020).

Although the results of anti-CD19 CAR T-cell therapy are quite satisfactory, there are some side effects or relapses. It is well established that lymphoma affects solid organs such as the lymph nodes, liver, and spleen. CAR T-cell therapy for lymphoma has been hampered by a number of common factors unique to solid tissues, such as factors in the tumor microenvironment, obstacles for CAR T-cells homing to tumor site, and also low tumor penetration between CAR T-cells and tumor cells.

The low transfection rate for the CAR T-cells has been reported due to individual factors which could be a part of the reasons for poor therapeutic effects (Bonati et al., 2015; Enblad et al., 2015; Filley et al., 2018). At present, a new type of CAR T-cell has been developed in Japan, which can penetrate into tumor tissues and induce a robust T cell and dendritic cell (DC) response and as well as play a synergistic antitumor role. It has been shown to achieve 100% curative effect in various tumor animal models; the clinical application of this technique may improve the efficacy of CAR T-cells in NHL (Adachi et al., 2018). In addition, there are many reasons for relapses: first, repeated antigen exposure can lead to T-cell exhaustion. Second, the mechanism of CD19-negative relapse may be attributed to the presence of CD19 blast primitive cells prior to the relapse. Under the repeated selection of anti-CD19 CAR T-cell therapy, CD19 cells develop as dominant clones and eventually lead to CD19-negative recurrence. Also, the deletion or alternative RNA splicing of exon 2 of chromosome 16, where the CD19 gene is located, resulted in the downregulation of B-cell transcription factors pair box 5 (PAX5) and early B-cell factor 1 (EBF1), which leads to lymphatic cell transformation to myeloid cell and recurrence (Fischer et al., 2017; Li and Chen, 2019).

Common toxicities of CAR T-cell therapy that have been observed include CRS, macrophage activation syndrome (MAS), and neurotoxicity (Grupp et al., 2013; Davila et al., 2014b; Frey and Porter, 2019; Murthy et al., 2019). Many cytokines released during CRS are found to be elevated, and the main cytokines related to the pathogenesis of CRS include IL-6, IL-10, IFN—γ, MCP-1, and GM-CSF (Martinez et al., 2009; Hunter and Jones, 2015; Tanaka et al., 2016; Wang and Han, 2018; Murthy et al., 2019). These toxicities can be self-limiting requiring only symptomatic care or may be treated with the anti-human interleukin 6 (IL-6) receptor monoclonal antibody and necrosis factor receptor type II antibody fusion protein and/or glucocorticoids (Murthy et al., 2019). The goal of treatment of CRS was to avoid toxicities and maximize the anti-tumor effect of cellular therapy. In the current study, fever, dyspnea, and shock were observed for CRS patient, and the IL-6 antagonist administered was obviously effective. When the effect of IL-6 antagonists and necrosis factor receptor type II antibody is inadequate, glucocorticoids are used. At the same time, fever, dyspnea, and shock can be alleviated in most cases after symptomatic treatment. Overall, these symptoms can be alleviated by reducing cytokines *in vivo* with IL-6 antagonists, necrosis factor receptor type II antibodies, or glucocorticoids. Therefore, we consider that fever, dyspnea, and shock are mainly attributed to CRS. Tocilizumab, a humanized IL-6 receptor antagonist mAb, works on both the membrane-bound IL-6 receptor and soluble IL-6 receptor by competitively competing with IL-6 for binding to both receptors, leading to decreased IL-6 signaling and reducing immune activation and inflammation (Nishimoto and Kishimoto, 2008; Kotch et al., 2019). It was approved by the FDA for the treatment of severe or life-threatening CAR T-cell-induced CRS in adults and pediatric patients ≥2 years old (FDA, 2017). Tocilizumab was later shown to reduce fever and CRS symptoms without affecting CAR T-cell levels in serum or bone marrow (Davila et al., 2014b). In addition, CRS can cause the rise of a variety of cytokines, including IL-6, TNF-α, and IL-10. We also try to use etanercept (Deeks, 2017) alone to reduce TNF-α to control CRS, which showed obvious effect. The glucocorticoids may be an alternative treatment for severe CRS and ineffective tocilizumab and/or etanercept treatment after CAR T cell therapy. CRS can also produce high levels of inflammatory factors and various cytokines, leading to life-threatening symptoms. When the patient’s vital signs are stable, the application of tocilizumab/etanercept or glucocorticoids can show significant curative effect regardless of the level of cytokines. However, we did not get the ideal effect when the high level of cytokines and vital signs were unstable. But, we observed that plasmapheresis has an immediate effect. CRS-related symptoms disappear as the high level of cytokines is controlled. Excessive inflammatory factors may also be controlled using extracorporeal blood purification techniques, such as high-volume hemofiltration, cascade hemofiltration, plasmapheresis, and coupled plasma filtration adsorption. The main objective of these techniques is to selectively eliminate high molecular from medium-weight components, such as cytokines (Lysenko et al., 2017). In addition, anakinra (Giavridis et al., 2018; Norelli et al., 2018) (inhibition of IL-1 binding to IL-1RI), dasatinib (Montero et al., 2011) (inhibition of T cell activation and T cell signal kinase), and lenzilumab (Teachey et al., 2016; Sterner et al., 2019) (GM-CSF antibody) may also be able to control CRS. Reducing and managing toxicity, as well as using CAR T-cell therapy in conventional clinical practice, still needs to be explored and resolved. CRS is the most common and potentially most serious adverse event after the reinfusion of CAR T-cells; if CRS can be effectively controlled, anti-CD19 CAR T-cell therapy is extremely safe and effective.

In our study, no correlation between cytokine levels and the severity of CRS cells were observed, which is consistent with the previous literature. The current research study also focuses to find cytokines that can accurately and effectively differentiate CRS response and to formulate stratified diagnosis and treatment strategies (Frey and Porter, 2016; Park et al., 2016; Hay et al., 2017; Neelapu et al., 2018) or to facilitate clinical detection indicators (CRP is currently recommended in many studies) (Davila et al., 2014b; Bonati et al., 2015). The detection trend of CRP in this study is similar to that of IL-6, which indicates that it can reflect CRS but cannot reflect the severity of CRS.

After CAR T-cell therapy, one MCL patient and one BL patient were given ibrutinib to maintain therapy and continuous CR for 41–42 months. The BTK inhibitor ibrutinib improves response of anti-CD19 CAR T-cell therapy in patients of MCL and reduces cytokine release syndrome (CRS). The ibrutinib and CAR T-cell derive additional synergy from ibrutinib-mediated T-cell mobilization and ibrutinib-mediated reduction in inhibitory receptor expression on CAR T-cells. Also, the killing of MCL cells by anti-CD19 CAR T-cells was significantly augmented in the presence of ibrutinib, suggesting an additive cytotoxic effect of the combination of both ibrutinib-sensitive (MCL-RL) and -resistant (JEKO-1) MCL cells. Therefore, ibrutinib and CAR T-cell may work in synergy for an enhanced antitumor effect (Ruella et al., 2016).

We noticed that IL-10 was significantly elevated in Case 2 with EBV-positive DLBCL. The BCRFL-coding frame of EBV is homologous to human IL-10, which is also known as viral IL-10. It has an immunosuppressive effect similar to IL-10. It has been reported that EBV and IL-10 work in synergy to promote tumorigenesis (Beatty et al., 1997; Liu et al., 1997; Irons and Le, 2008; Samanta et al., 2008; Davis et al., 2010). Hence, CAR T-cell therapy is both effective and safe. We will continue to improve the prospect of oncology to ensure that such therapy can be safely administered to all patients.

## 6 Conclusion

CAR T-cells are not only effective and safe in the treatment of R/R B-cell lymphoma, but they are also effective in the treatment of MRD of invasive B-cell lymphoma. CRS is the most common and serious side effect in the course of CAR T-cell treatment. When symptomatic treatment of CRS fails to give satisfactory results, CRS can be controlled by the IL-6 receptor antagonist. In the event that adverse effects remain uncontrollable after the administration of the IL-6 receptor antagonist, glucocorticoids and/or plasmapheresis can be administered for the treatment of CRS.

## Data Availability

The original contributions presented in the study are included in the article/Supplementary Material; further inquiries can be directed to the corresponding author.
